# Supplementary material for: Upconversion Nanoparticle-Based Fluorescent Film for Distributed Temperature Monitoring of Mobile Phones’ Integrated Chips
Source: Nanomaterials (Basel). 2023 May 23;13(11):1704. doi: 10.3390/nano13111704 (PMC10254155; doi:10.3390/nano13111704)
Supplement: Supplementary file 1 [file nanomaterials-13-01704-s001.zip › nanomaterials-2400309-supplementary.pdf]

Electronic Supplementary Material (ESI) for Nanomaterials.
This journal is MDPI

Supporting Information *for*

Upconversion nanoparticle-based fluorescent film for distributed temperature monitoring of mobile phone integrated chips

Hanyang Li *, Miao Yu, Jichun Dai, Gaoqian Zhou and Jiapeng Sun

College of Physics and Optoelectronic Engineering, Harbin Engineering University, Harbin 150001, China.

*E-mail: hanyang_li@hrbeu.edu.cn

Contents

1. Figure S1. HRTEM image, TEM image and size distribution of $NaYF_4$:Yb/Er@$NaYF_4$........ S-2

2. Figure S2. The element maps of UCNPs/PDMS. S-2

3. Figure S3. The preparation process of the optical film S-3

4. Figure S4. Schematic diagram of fluorescent film temperature sensing device........................ S-3

5. Figure S5. FIR values at different positions of the film.. S-4

6.Figure S6. The temperature sensitivity of fluorescent film with the thickness of ≈23μm S-4

7. Figure S7. The temperature sensitivity of fluorescent film with the thickness of ≈67μm......... S-5

8. Figure S8. The temperature sensitivity of fluorescent film with the thickness of ≈80μm S-6

9. Figure S9. Chip temperature monitoring setup diagram S-7

10. Figure S10. Distributed temperature measurement at the interval of 10μm S-7

11. Figure S11. The real-time changing spectrum of the chip center points collected of the first 200s. S-8

12. Figure S12. The chip surface temperature measured was MediaTek 6752, Huawei Qilin 970, and Apple A10.. S-9

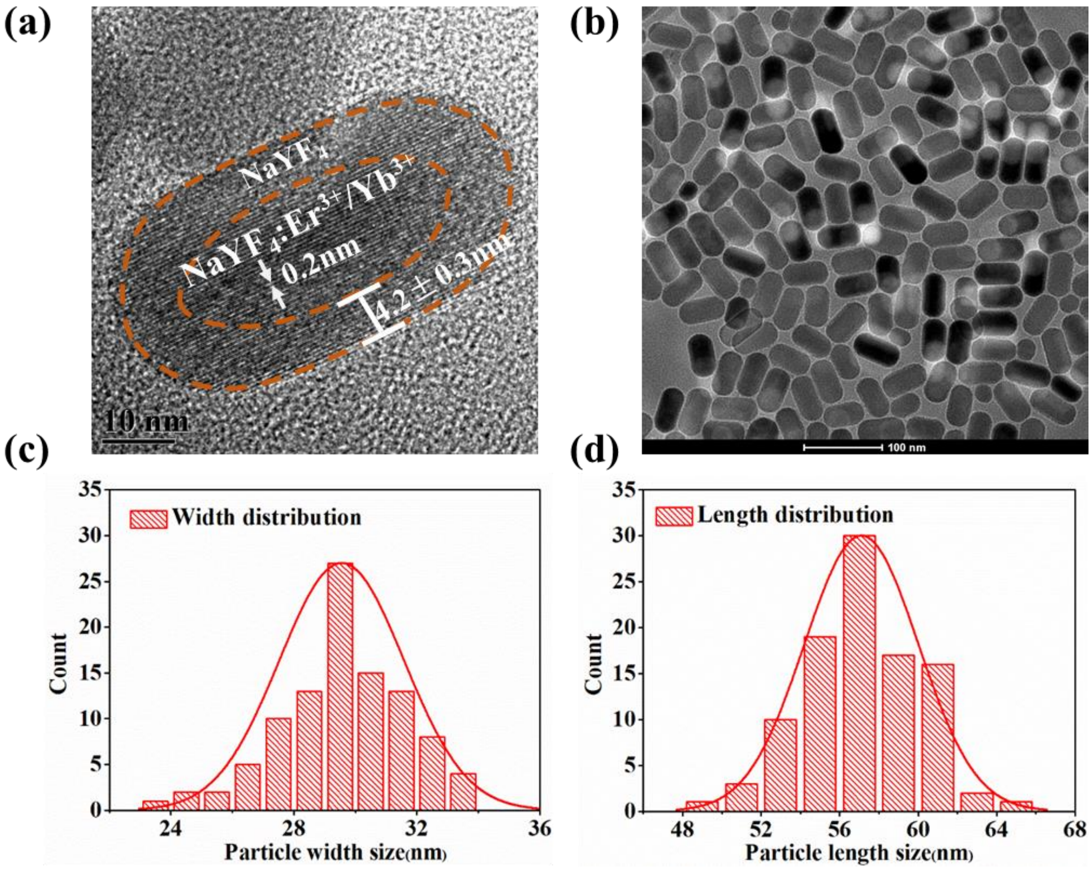

Figure S1 (a) HRTEM image and (b) TEM image of $NaYF_4$:Er^{3+}/Yb^{3+}@$NaYF_4$. (c and d) The size distribution charts of $NaYF_4$:Er^{3+}/Yb^{3+}@$NaYF_4$.

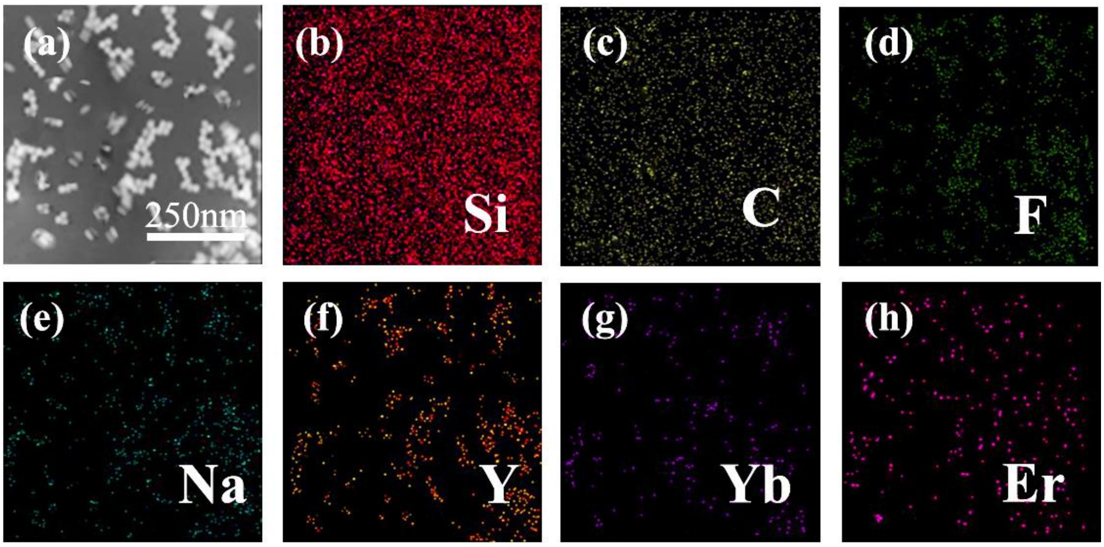

Figure S2 (a) TEM micrographs of NaYF4:Er3+/Yb3+@NaYF4/PDMS. (b–h) The element maps for Si, C, F Na, Y, Yb, and Er of NaYF4:Er3+/Yb3+@NaYF4/PDMS.

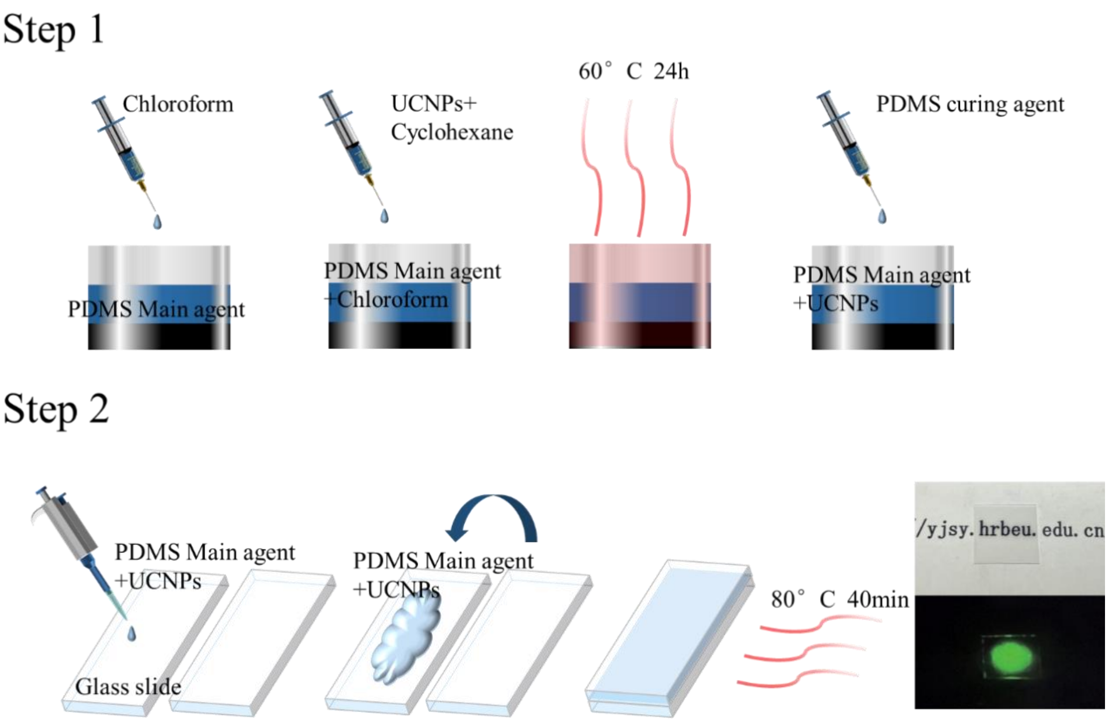

Figure S3 Step 1: Preparation of the UCNPs/PDMS. Step 2: Fabrication of the film.

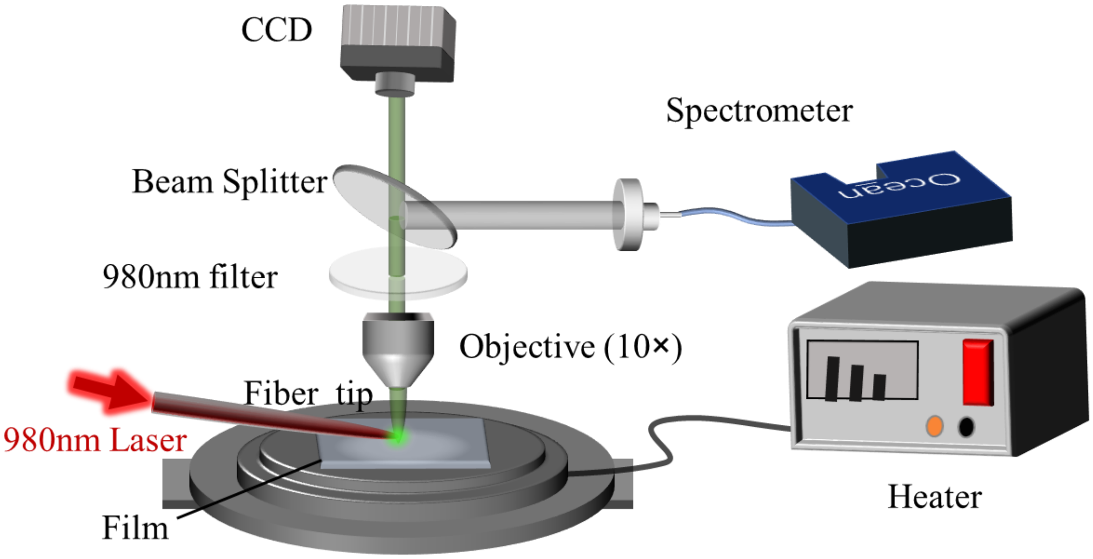

Figure S4 Schematic diagram of fluorescent film temperature sensing device based on UCNPs/PDMS composite.

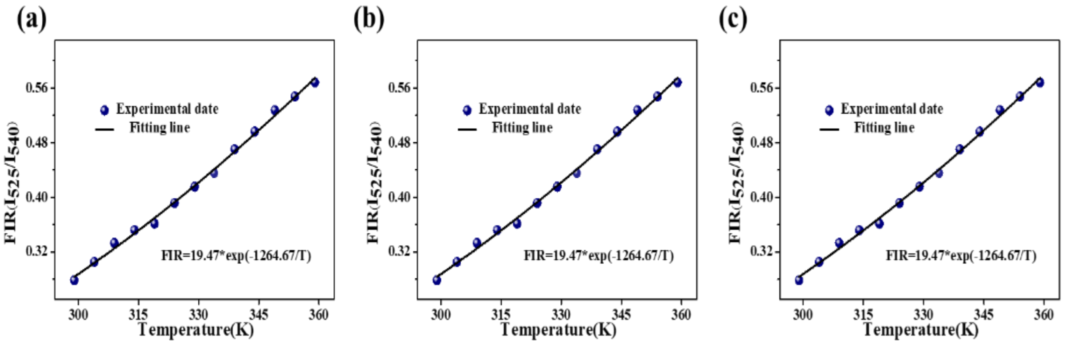

Figure S5 (a–c) Fitting curves between FIR values and temperature at different positions of the film. Randomly select three information collection points for the same film to avoid deviations and limitations.

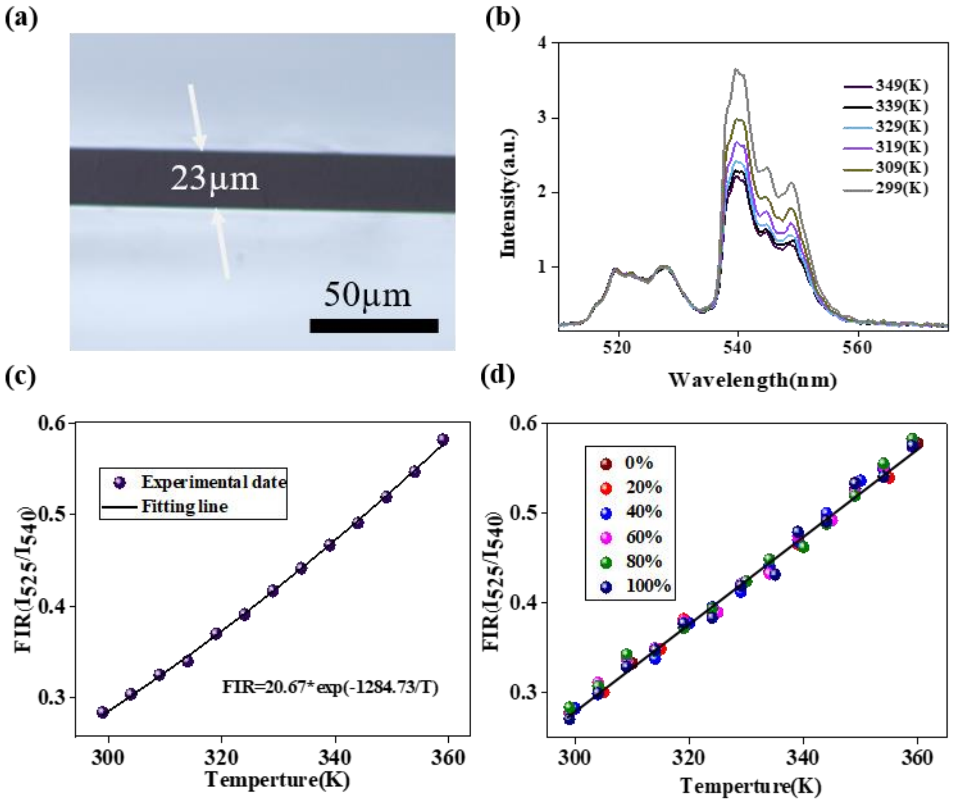

Figure S6 (a) The image is a photograph of the fluorescent film of 23μm. (b) The variation of emission spectra of the thermometer under temperature changes in the range of 299–349 K. (c) FIR versus temperature. (d) Dependence of FIR(I_{525}/I_{545}) on the temperature (K), measured under various tensile strains up to 100%.

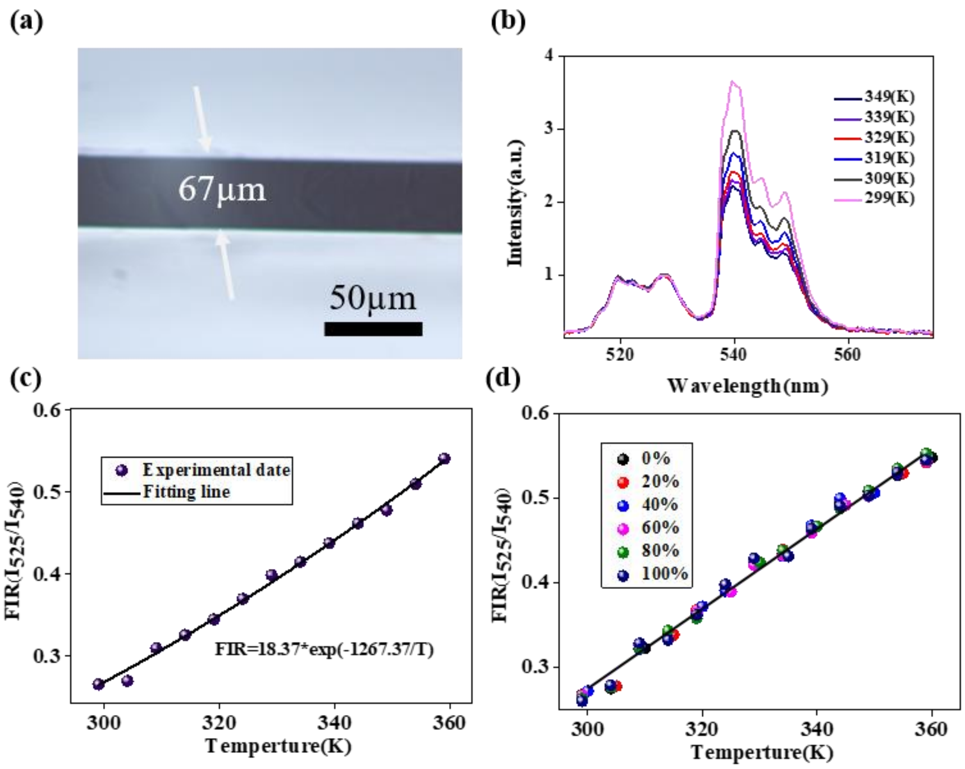

Figure S7 (a) The image is a photograph of the fluorescent film of 67μm. (b) The variation of emission spectra of the thermometer under temperature changes in the range of 299–349 K. (c) FIR versus temperature. (d) Dependence of FIR(I_{525}/I_{545}) on the temperature (K), measured under various tensile strains up to 100%.

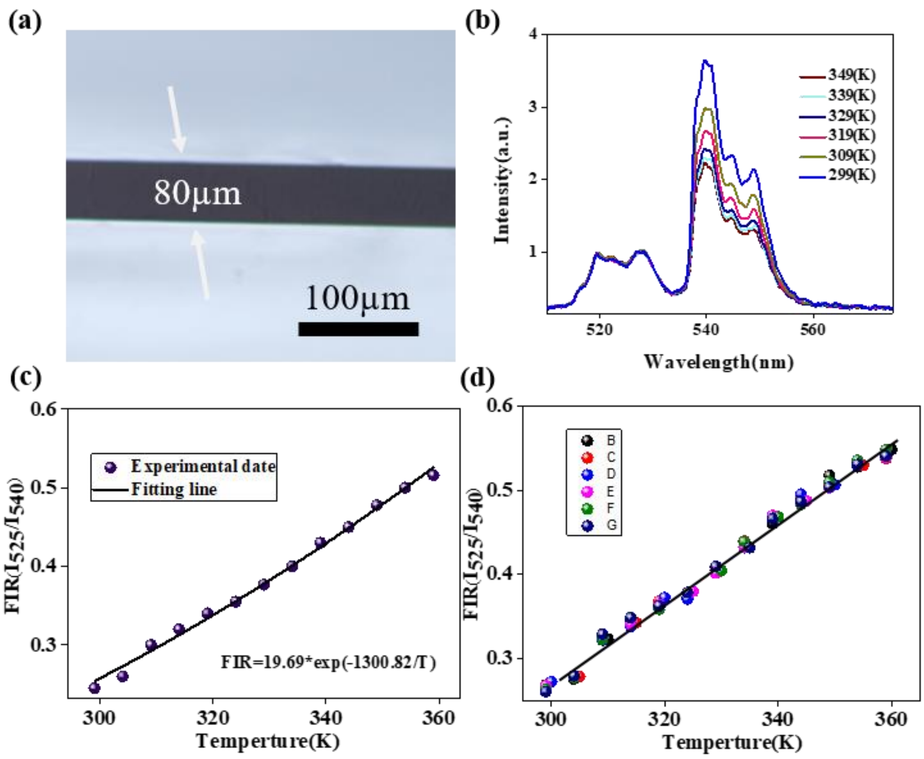

Figure S8 (a) The image is a photograph of the fluorescent film of 80μm. (b) The variation of emission spectra of the thermometer under temperature changes in the range of 299–349 K. (c) FIR versus temperature. (d) Dependence of FIR(I_{525}/I_{545}) on the temperature (K), measured under various tensile strains up to 100%.

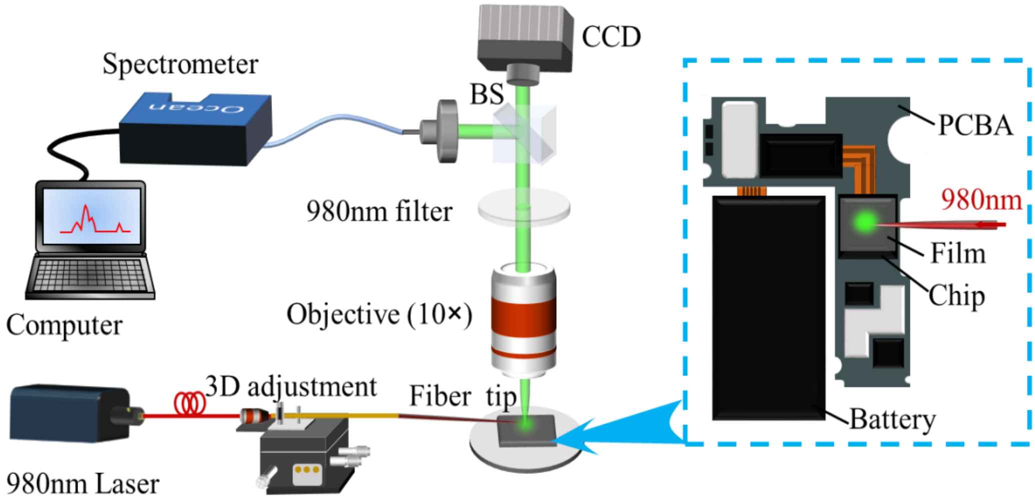

Figure S9 Schematic diagram of the chip surface temperature sensing device based on UCNPs/PDMS composite optical film temperature measurement.

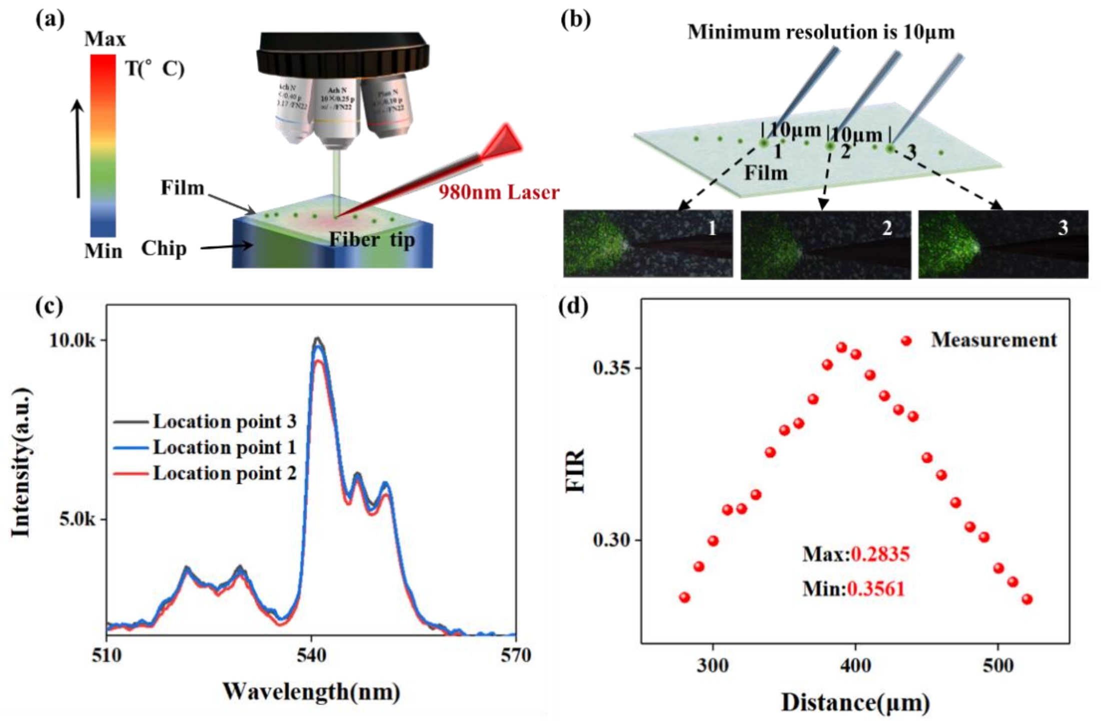

Figure S10 (a)Schematic diagram of the experimental setup for temperature measurement. (b) A simple schematic diagram of film temperature measurement and the inset is a microscope image for Er^{3+}-doped film. (c) Upconverted fluorescence spectrum image of location point 1、2 and 3 in b. (d) Photographs of the relationship of actual measured FIR and guiding distance.

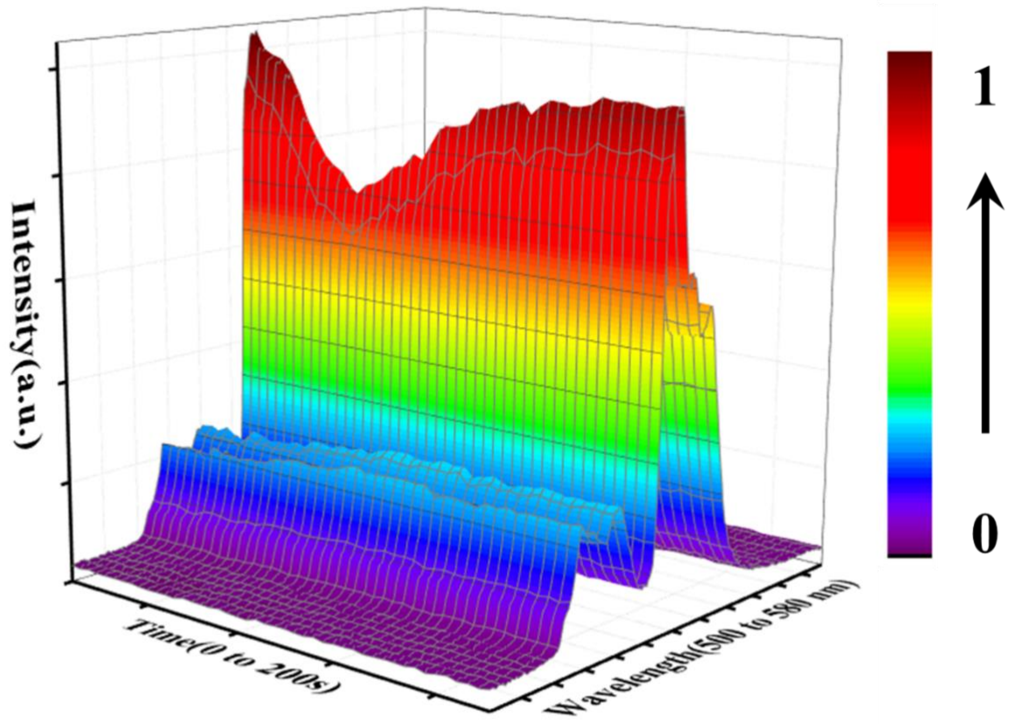

Figure S11 The real-time changing spectrum of the chip center point collected every 5s, the wavelength range of the first 200s is 500 nm~580 nm.

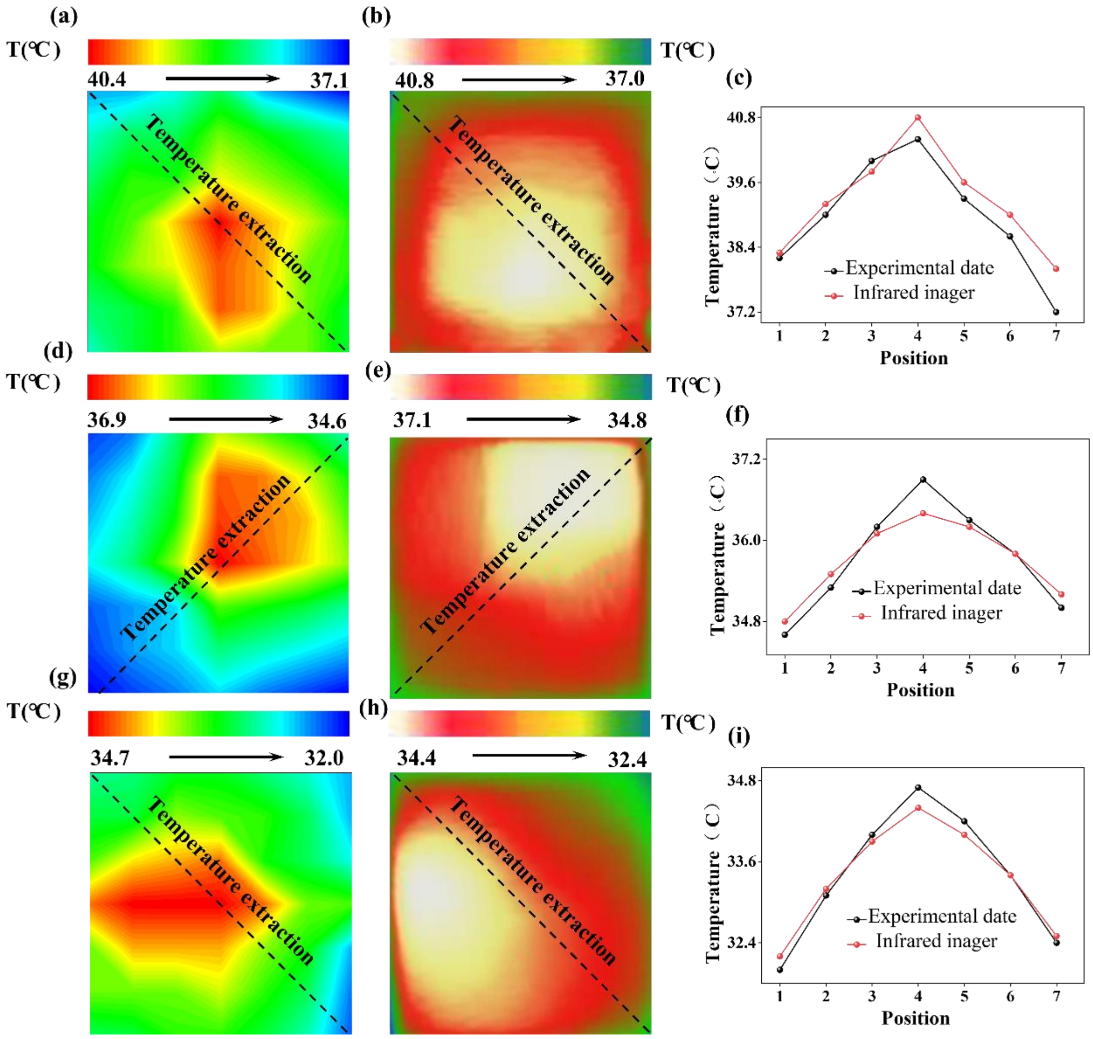

Figure S12 The above figure shows the chip surface temperature (a, d, g) measured by MediaTek 6752, Huawei Qilin 970, and Apple A10 and the infrared thermal imaging (b, e, h) of the tested chip. Fig. (c, f, i) comparison between the measured temperature of the film on the chip surface and the measured temperature of infrared thermal imaging.
